# Creating a Framework for Online Cancer Services Research to Facilitate Timely and Interdisciplinary Applications

**DOI:** 10.2196/jmir.7.3.e34

**Published:** 2005-07-01

**Authors:** Pamela Whitten, Gary L Kreps, Matthew S Eastin

**Affiliations:** ^3^School of CommunicationOhio State UniversityColumbusOHUSA; ^2^Department of CommunicationGeorge Mason UniversityFairfaxVAUSA; ^1^College of Communication Arts and SciencesDepartment of Telecommunication, Information Studies and MediaMichigan State UniversityEast LansingMIUSA

**Keywords:** Online information services, cancer communication, health outcomes, behavior change, cancer survivorship

## Abstract

Researchers from a wide array of disciplines have conducted engaging and informative studies in recent years concerning the use of the Internet for cancer-related services. Typically, these publications provide key data related to utilization statistics, how online information can be used, what users want or expect from the Internet, outcomes or impacts, and quality and credibility of websites. These are important themes for understanding online cancer issues. However, this special issue of the Journal of Medical Internet Research seeks to recast these themes in a way that will facilitate pragmatic and applied means of employing data in prescriptive and interdisciplinary ways. This issue includes 14 papers that exemplify applications for the research framework recommended in this paper. This framework includes an expanded focus on the development and design of online cancer services, online consumer behavior/communication, behavior change, and living with cancer.

## Introduction

Cancer is a serious, complex, and frightening set of diseases that demands effective communication from health care consumers and providers [1]. A cancer diagnosis is a life-changing and personal event. Increasing access to relevant information technologies, such as the Internet, has changed how individuals learn about, treat, and live with cancer [2], as well as how physicians learn about, treat, and interact with cancer patients [3]. The papers in this special issue of the Journal of Medical Internet Research examine the development and use of important online cancer services, and this editorial helps to frame fruitful directions for research on online cancer information services.

Generally speaking, 80% of US adult Internet users, or 73 million Americans, have searched the Web for at least 1 of 16 major health topics [4]. Health care websites are among the most visited sites online [5]. That said, while many people use the Internet as a health information resource, patients with cancer have been identified as particularly high users of the Internet for information about their disease, treatment, life after cancer, and health care providers [6]. Researchers evaluating the public's use of the Internet [7-9] have concentrated on the Internet's ability to reach various populations. Fogel et al, for example, noted that those seeking breast cancer information on the Internet tend to be well educated and wealthy, and non-white consumers were less likely to seek information about breast cancer than white health care consumers [8].

While the Internet offers great opportunities for both patients and physicians, many oncologists believe that the Internet is an information source that can make patients hopeful, confused, anxious, and knowledgeable [10]. Stimulated by the potential to redefine how patients and physicians deal with cancer-related illnesses, researchers from various disciplines have begun to investigate the Internet and its potential role in cancer research and treatment. To this end, scholarly activity regarding online cancer services is thriving. A 2005 key term search of Medline over the past 6 years (key term “cancer and Internet”; search conducted January 2005) yielded 808 citations. Though this paper does not represent a formal content analysis of these 808 publications, a review of the publications from the past 5 years points to a plethora of activity.

This paper will provide an overview of the contributions of recent studies on online cancer services that focus on utilization, information use, individual goals, and outcomes. As important as these issues are, the purpose of this special issue is to expand our understanding of online cancer services into categories that offer immediate prescriptive information and facilitate the employment of interdisciplinary strategies. This paper concludes with recommendations about conducting translational cancer information services research and provides an overview of the papers in this special issue.

## Current Online Cancer Research

A number of current research papers examine utilization statistics for online health care and cancer services, often with mixed results. For example, in 2003, Eysenbach and Kohler [11] reported that 4.5% of all online searches are health related, and those 6.75 million health-related searches are conducted online every day. Eysenbach [12] performed a meta-analysis of 24 published surveys and estimated that, in the developed world, almost 40% of persons with cancer are using the Internet. In a 2002 publication, Mills et al [13] conducted a survey in which patients most frequently cited the hospital consultant, general practitioner, and chemotherapy/radiotherapy staff as sources of information. The Internet was employed by less than 10% of the 430 patients in this study. Other publications invite further detailed analysis of actual utilization by disenfranchised populations. For example, Fogel [8] reviewed cancer literature regarding Internet health information use among diverse racial/ethnic populations and low literacy groups. He found 8 relevant articles and concluded that little empirical research existed concerning the online practices of racial/ethnic and low literacy groups.

Many researchers have contributed to knowledge in this field by studying the ways that online information is used. Eysenbach [12] reported four areas of Internet use: communication (email), community (virtual support groups), content (health information), and e-commerce (purchase of goods or services). Often, formal research is conducted to document whether cancer patients actually utilize content-specific materials available online. In a study of patients attending a Midwestern US lung cancer clinic, only 16% actually used the Internet to gather information, even though 60% expressed interest in using the Internet [14]. A recent qualitative study of 175 men and women conducted in the United Kingdom found that cancer patients used the Internet for a wide range of informational and support needs through all stages of cancer care, from early opinions to follow-up after treatment [6]. A study of 295 men undergoing radiotherapy for prostate cancer found that a significant number of men used the Internet for information; however, even with Internet access in the home, other factors such as race may impact Internet use [15]. Other studies have delved specifically into factors that may impact use of the Internet for cancer information. For example, a study by Bowen et al [16] found that predictors of use for a breast cancer Web-based intervention included employment, perceptions of health, and mental health scores. Other research interventions suggest that use of the Internet for cancer-related services may work best when formal training is offered to cancer patients. Edgar et al [17] found that subjects who learned to access relevant Internet sites through one-on-one teaching sessions with a medical librarian expressed more confidence in their perceived ability to evaluate the information.

Other publications to date have addressed what people want from the Internet in relation to online cancer services. These studies delve into the specific information needs of cancer patients, such as the study conducted by Rozmovits and Ziebland [18]. These researchers explored the information needs of cancer patients and sought to determine if a specific website (DIPEx) would have addressed specific unmet information needs of people with breast or prostate cancer. Education is a common theme regarding desired applications. Brooks [19] provided an overview of the evolution of patient education on the Internet, reviewed the Patient and Family Education Standards of the Joint Commission on Accreditation for Healthcare Organizations (JCAHO), and offered guidelines for nurses wishing to use the Web for patient education. Other papers provide examples of nontraditional expectations for online cancer services. Eysenbach and Wyatt [20], for example, called for multiple uses of the Internet in the research process, from identifying research through using the Web for surveys and clinical trials to using the Web to publish research.

Another theme found in the current online cancer literature concerns outcomes and impacts. Nguyen et al offer a good review of studies that have evaluated the impact of specially designed Internet-based programs [21]. They conclude from their review that some outcomes in certain groups can be moderately improved and that overall user satisfaction is positive. Other studies in this area seek to increase understanding of the impact of online information on medical care. Pereira et al found that more than 60% of patients who had used the Internet to gather information were seeking treatment options or alternatives beyond those offered by their physician [22].

Finally, a large number of publications regarding the Internet and cancer focus on the quality and credibility of existing websites. Many publications express concern about the staggering amount of health information available online and suggest pragmatic ways for consumers to cope with complex and often contradictory online health information. Several publications document the challenges consumers face in evaluating the quality of information provided by typical searches [23] and highlight major sites with credible information [24]. In one case, researchers identified four potential red flags consumers can use for evaluating the quality of online cancer information sites: availability of online purchasing, inclusion of patient testimonials, description of the treatments as cancer cures, and description of the treatments as having no side effects [25]. Other authors offer general categories for evaluating cancer websites, such as examining Web content, usage, authorship, and publications [26]. Hoffman-Goetz and Clarke concluded that there is great variability in Internet breast cancer sites with respect to the framework criterion of accountability and that many sites omit fundamental indicators such as dating and sources [27].

Publications to date for online cancer services have provided crucial pilot data and editorial input. Yet, they often have a narrow focus that does not factor in alternative factors or explanations. More importantly, it is too easy to oversimplify the contribution of research to date with such a focus on utilization, use, and quality. These are crucial issues that merit expanded study. We argue that this research can and should be performed in the context of relevant studies that offer immediate impact on the lives of cancer patients, their caregivers, and health providers. As a result, we encourage expansion of online cancer research in four pragmatic and applied categories.

## An Expanded Framework for Applied Online Cancer Research

There is strong consensus among researchers of online cancer services that study results should have a timely impact [1,2]. To promote the translation of online cancer communication research into practice, we propose an expanded research framework that emphasizes (1) development and design, (2) online activities and communication, (3) behavior changes, and (4) living with cancer.

### Development and Design of Online Cancer Services

The Internet provides a unique and powerful channel for providing relevant cancer-related health information and services to those confronting cancer (consumers, providers, and advocates). Accessing information online, or becoming skilled at “navigation,” represents the first step toward effective utilization of online information [28,29]. Traditionally, navigation refers to moving through space; however, navigation through cyberspace entails “virtual movements through cognitive space made up of data and the knowledge emerging from those data” [30]. Information providers who fail to provide user-friendly sites that are easy to navigate [31,32] may create websites that are perceived as disorganized, confusing, and frustrating. An important component of navigation is the extent to which searches bring users to the information sought, or the search “hypertext efficacy.” General search engines explore the entire Web, whereas directories just search sites that have been classified and indexed by that directory. Both search engine and directory users seek information by typing in key words or phrases of interest.

In addition to examining who uses online information services, it is also important to determine where and how users are going online [33]. Research in this area points to important issues such as access to telecommunication services and hardware availability [34,35]. Hardware can mean access to a conventional PC; however, wireless and mobile technologies are transforming this concept to include handhelds and even cellular phones.

Research on the development of online services often provides important information regarding information utilization and access, but such research might fruitfully provide relevant data about the sources of information. Web development does not always mean creating content from scratch. Indeed, as the Internet becomes more sophisticated, development often means creating means of accessing extant credible information sources such as the Physician Data Query (PDQ) database, designed by the National Cancer Institute, which provides an important cancer therapy database to wired physicians as well as to cancer consumers and advocates [36].

Design elements are uniquely important for Internet-based services. The way a website is designed impacts a user's ability to initially search and find the site; successfully navigate the site; understand, use, and retain information from the site; perceive high levels of efficacy; and judge the site to be credible and useful. The design of a website also impacts further use of the site and the Internet in general. For example, Fogg et al [31] indicate that sites that make sense to the user and are easy to navigate and are perceived as credible. Further, it has been argued that the dynamic nature of a website (eg, advertising, colorful animation) acts as noise to the central content, thus making it difficult for users to retain site information [31,37,38]. The bottom line is that design has a crucial impact on who comes to a site in the first place, how the site is employed, and whether it successfully accomplishes the goals of its creators.

### Online Activities and Communication

Online cancer-related activities include searching for information, participating in online communities, and even purchasing health-related goods and services. The multiple functions of online cancer services beg for research that explains how people behave and interact online. How do we explain communications and interactions that occur during online activities?

Preliminary research provides important hints about how people act online and the unique ways in which they communicate with one another. For example, important partnering activities have become an important feature of online cancer information systems. The Association of Cancer Online Resources (ACOR), for example, serves as a one-stop mailing list resource for various kinds of cancer. ACOR monitors and maintains more than 70 mailing lists and has more than 76000 subscribers, with a goal of offering users the latest and most accurate health information [2]. More than 100 volunteers actively review ACOR content to assess and ensure information accuracy. This represents an important community for both accessing and monitoring cancer information.

Often, online cancer services are used in conjunction with in-person care. New lines of research need to inform how these two services are integrated and in what ways they are discrete or iterative. In a survey of more than 500 patients, Diaz et al found that, of those using the Internet for information, almost 60% did not discuss these searches with their doctor [39]. Interestingly, discussion of this information with a doctor did have an impact. Patients who did discuss this information with their physician rated the quality of the online information as high. It would seem undeniable that the Internet is becoming a third party in the doctor-patient relationship. We may find that health professionals must become as proactive as their patients when it comes to online services. In a Colorado-based study, researchers found that patients were interested in getting email reminders about appointments, booking online appointments in real time, and receiving updates about new advances. They also desired virtual visits for simple or chronic problems [40]. In some cases, virtual care seems to adequately address needs. The Oncology Nursing Society, for example, launched an interactive, confidential Internet resource where cancer patients and caregivers can have their questions about cancer fatigue answered quickly.

Understanding online communication behaviors requires a complete understanding of communication practices and preferences. As intriguing as the rapid growth of online health communication has been, it is often tempered by consumer preferences for more traditional forms of health communication. A recent study by Basch et al concluded that, despite the great attention paid to understanding the quality and usability of online cancer content, print health communication products remain the most common source of information sought by patients with cancer [41].

Yet, anecdotal evidence indicates that online opportunities are changing people's lives. Cancer patients have talked about the Internet saving them spiritually and psychologically by enabling them to do things like compare notes with patients around the world [42]. A wide range of online sites allows users to participate in email discussions groups and connect directly to cancer treatment sites, medical journals, news articles, and cancer survivors. Communication online is a rich tapestry of individual interactions with information and interpersonal or group discussions and support. When people enter the online world, they can simultaneously partake in multiple behavioral strategies and relational dimensions. Research that facilitates an understanding of the richness of these interactions and relationships will offer great benefit to many stakeholders.

### Behavior Change

Another important area for investigation involves careful examination of the goal of many online cancer sites to influence health behaviors that can help prevent disease, promote health, increase treatment efficacy, and enhance quality of life [1]. Preventive health specialists are particularly intrigued by the potential of online health services to modify risky behaviors. The contribution and impact of mediated communication such as the Web merits significant research. A study by Mullen et al pointed to the potential importance of online information sources for health education and risk prevention [43]. They concluded that online media plus personal communication can produce significant influences on smoking, alcohol, nutrition, and weight-control behaviors. A host of health organizations are now using the Web as a tool to manage client health behaviors. HealthPass members, for example, participate in an initial health risk assessment and are then directed toward online lifestyle management programs to meet their individual needs. The InternMountain Health Care offers a Preventive Health Online Center, which directs users to an appropriate health care decision after they identify their symptoms. The Self-Management @ Stanford Healthier Living with Ongoing Health Problems is an online workshop (and study) given on the Internet. Here, people with heart disease, lung disease, or type 2 diabetes participate together. However, this online workshop is designed to enhance regular treatment and disease-specific education. In addition to attempting to modify general risk behaviors, online tools also are being employed to maximize the effectiveness of behaviors that impact successful treatment for those diagnosed with cancer. Fleisher et al, in a study of 500 patients who were newly diagnosed with cancer, found a significant relationship between Internet use and perceived patient task behavior and self-efficacy [44].

Research concerning the actual impact of online cancer services on modifying behavior is in its infancy. It would seem that we need to look at some of the pioneering work conducted in the 1990s that hints at important questions that must be addressed in order to explain these successes and failures. For example, Mandelblatt and Yabroff pointed to the priority of designing interventions to target providers rather than the patients [45]. These researchers found that interventions targeting both patients and providers were not significantly better at increasing mammograms than those targeting providers alone.

It is crucial that research that documents the successes *and* failures in impacting behavioral change through online interventions is disseminated as rapidly as possible. The clock is ticking for those currently engaging in risky behaviors and for those whose treatment success could depend on modified behavior.

### Living With Cancer

The diagnosis of cancer is not a death sentence. In fact, there were almost 10 million cancer survivors in the United States in 2001 (data was collected from 1971 to 2001). Further, estimates suggest that 1 of 6 people over the age of 65 is living with a history of cancer [46]. The Web offers an important source of relevant health information for cancer survivors [47]. Current research often applies a narrow lens for examining online information as a key tool only for those diagnosed with cancer, while it is also a key resource for those living with cancer. The National Cancer Institute's website offers crucial information on the role of cancer trials in advancing cancer research and is aimed at both patients and providers. Findings from a study examining how patients participating in cancer clinical trials perceived and used electronic communication underscored the desire of patients to communicate with others in the same clinical trial, as well as with their health care providers, via the Internet [48].

However, other online services are also being developed for those coping with the effects of cancer and its treatment on a daily basis. One of the most exciting online activities falls within the rubric of support services. Data from traditional support services indicate that education, physician referral, social support, and spirituality may be important influences on the use of cancer support services [49]. Are there variations in these predictors as cancer patients and caregivers move into virtual support communities? Some studies suggest that certain patient traits may influence the likelihood of participating in an online support group. For example, Klemm and Hardie provide data that suggest that cancer patients who are depressed prefer to use Internet support groups rather than in-person, face-to-face support groups [50]. A review of 9 research articles by Klemm et al [51] concluded that online cancer support groups helped people cope more effectively with their disease, though the authors caution that the papers are riddled with methodological challenges. In regard to general coping issues, Fogel [52] cautions that Internet health information use is not associated with psychological coping in breast cancer patients. However, other work indicates that Internet use by people with cancer may serve to restore self-image [6].

Of particular interest in this category is the convergence of health care and the Internet. Frank [53] defines pure digital health care companies as falling into one of three areas, namely content, connectivity, and commerce. Research that examines the Internet as a commercial means to purchase goods and services is almost nonexistent at this point. However, more and more patients are turning online to purchase traditional medications and supplies and to seek alternative treatments.

The expanded research framework we propose for the study of online cancer services in pragmatic and interdisciplinary ways does not exclude the need to address traditionally framed research questions such as those discussed in the first section of this paper. However, we encourage researchers to conduct studies that also examine the four proposed applied categories that we believe can promote the translation of online research into enlightened cancer information practices, including understanding issues related to the development and design of cancer websites, strategies for interacting and communicating effectively online, ways to use online services to influence relevant health behaviors, and the use of online services for supporting the information needs of cancer survivors. The final section in this editorial provides an overview of the papers in this special issue, and it suggests how these papers illustrate a research focus on these four applied categories.

## Overview of This Special Issue

The papers in this special issue illustrate the rich opportunities available to expand online health communication inquiry to examine the development and design of online cancer services, to understand the ways information users communicate online, to track the influences of online services on relevant health behaviors, and to evaluate the information needs of cancer survivors.

### Development and Design of Online Cancer Services

Patrick et al [54] provide a thought-provoking paper that breaks the mold concerning assumptions about the development and design of online services. Specifically, these authors employ an ecological theoretical perspective to explain the need to understand the highly complex relationships between and among individuals, society, organizations, the built and natural environments, and personal and population health and well-being. Developing interventions solely based upon individual psychosocial and cognitive processes offers limited strategies to develop Internet-based resources to reach individuals across all the domains of cancer, including prevention, early detection, treatment, survivorship, and end-of-life care. Eng [55] moves development beyond the traditional notion of the content. Instead, he argues that accelerating the application and deployment of emerging technologies to population health change requires a multifaceted approach, including transdisciplinary intervention programs, increased funding, facilitative infrastructure, and policy changes. LaCoursiere et al offer a sample of prescriptive information that has important implications for content development and website design [56]. Their study analyzed cancer patients' attitudes toward five dimensions of online health care, including community and news, trusted information and advice, disclosure, self-efficacy in evaluation, and outcomes. Grama et al [57] present an overview of the National Cancer Institute's (NCI's) multipronged approach to gathering input about its online information products—using stakeholder meetings, focus groups, standard and customized online user surveys, usability testing, heuristic reviews, and search log analysis. The authors highlight some of the many enhancements that have been made to NCI's online cancer information products based on user input.

### Online Activities and Communication

Rimer et al [58] offer an engaging and applied analysis of how consumers employ a specific type of online cancer-related support—online mailing lists. This paper provides insightful and specific detail regarding communication interactions, both information seeking and supportive communication, from this important online resource. Walther [59] et al provide a comprehensive overview regarding sociotechnical attributes related to online discussion systems, such as interactivity, presence, homophily, social distance, privacy, and interaction management. This paper offers a plethora of examples to illustrate how these concepts impact the ways users communicate and interact via online media. Wood et al [60] provide a microfocused examination of the link between usage data and market space. In their paper, they apply Internet audience measurement methodology to develop estimates of the positions of the National Library of Medicine and the National Institutes of Health in health information market sectors. Such analyses offer important contributions as we increase our understanding of the impact of “location” on online health services. Consumer health interactions and communication are impacted by virtual geography, and this paper provides a first look at this proposition. Metz et al address how many patients access the Internet to obtain cancer clinical trials information [61]. They provide specific analysis of OncoLink, the Internet-based educational resource managed by the University of Pennsylvania Cancer Center. Their report shows how a significant number of patients use the Internet for finding clinical trials. Cooper et al examine the interrelations of cancer-related search engine use with media coverage of cancer issues [62]. They studied Yahoo! search activity related to the 23 most common US cancers and found that search activity associated with specific cancers correlated both with the estimated incidence of these cancers and with specific news coverage about the cancer. This study illustrates that online cancer information search activities do not occur in isolation of other forms of communication and indicates the importance of analyzing online communication within the broad multichannel media environment.

### Behavior Change

Evers et al argue that researchers must examine the quality and effectiveness of online programs available to the general public in order to enhance predictive knowledge about population readiness to participate in such programs and implement behavioral changes [63]. These authors provide basic screening and extended evaluation criteria as templates to be used by developers and consumers to broaden behavior change knowledge beyond the typical early adopter. Graham and Abrams [64] employ a macro approach to their work by advocating for strategies to disseminate effective behavioral science interventions via the Internet to decrease risky health behaviors. They call for transdisciplinary approaches to promote lifestyle change across the cancer continuum, from primary prevention to treatment to survivorship.

### Living With Cancer

Bradley [65] offers an important and innovative paper that addresses use of the Internet to find information regarding the consequences of cancer diagnosis, treatment, and survivorship. Bradley concludes that patients can find information on sources of financial assistance, but cannot estimate the cost of their care or anticipate the impact cancer and its treatment may have on their jobs. The implications from this analysis provide important prescriptions for assisting those living with cancer to become informed consumers and skilled negotiators. Doolittle and Spaulding [66] offer a comprehensive review of the types of services offered online for cancer survivors, linking these services to increased cancer awareness, prevention activities, and actual documented health outcomes. This pragmatic paper grounds us in the ongoing need to pair the use of online cancer services with actual outcomes and impacts. Nguyen et al [67] examined the use of two websites developed for people living with cancer—the People Living with Cancer website from the American Society of Clinical Oncology and the Breast Cancer Info website from the Susan Komen Breast Cancer Foundation. Study participants were multiethnic, multilingual cancer patients at a public county hospital. They found that these diverse cancer survivors made good use of both websites and found the cancer information on the sites to be both understandable and useful. This study illustrates the utility of online cancer information services for a diverse audience of cancer survivors.

## Conclusion

This special issue offers intriguing samples of research that illustrate the importance of an expanded applied framework for online cancer communication inquiry. Our goal is to help researchers frame their inquiries to minimize the time from study conclusion to impact on the provision of online cancer services, facilitating the translation of health communication science into practice. This issue is only a first step. An edited volume is being prepared to expand this framework by incorporating additional papers for each theme. In addition, the book will expand the applied research framework presented here by advancing interdisciplinary strategies to conduct online health communication research.

## Editorial Note

Papers 54, 55, 58-60, and 63-66 were invited contributions solicited, peer reviewed, and edited by the guest editors of this issue, with further edits made by the JMIR editor. Papers 56, 57, 61, 62, and 67 were contributions submitted in response to an open call for papers or were regular JMIR submissions, which underwent the regular JMIR peer-review process and were edited by the JMIR editor.
